# The role of facet joint degeneration in the treatment success of transforaminal epidural steroid injection: a retrospective clinical study

**DOI:** 10.1007/s00256-025-04868-8

**Published:** 2025-01-22

**Authors:** Merve Sekizkardes Tutuncu, Savas Sencan, Canan Bilekyigit Kurt, Serdar Kokar, Osman Hakan Gunduz

**Affiliations:** 1 Department of Physical Medicine and Rehabilitation, Division of Pain Medicine, Marmara University Pendik Training and Research Hospital, Istanbul, Turkey; 2 Department of Physical Medicine and Rehabilitation, Marmara University Pendik Training and Research Hospital, Istanbul, Turkey

**Keywords:** Transforaminal epidural steroid injection, Pain, Facet joint degeneration

## Abstract

**Objective:**

Transforaminal epidural steroid injection (TFESI) is highly effective in alleviating radicular back pain. While predictive factors for TFESI treatment outcomes have been previously studied, there is a lack of data on the relationship between facet joint degeneration and TFESI efficacy. This study is aimed at studying the impact of facet joint degeneration on TFESI treatment outcomes for unilateral radicular pain.

**Design:**

A retrospective analysis was conducted on patients with unilateral radicular pain who underwent lumbosacral TFESI. Pain severity was assessed using the Numerical Rating Scale (NRS) at baseline, 1 h post-procedure, and 3 weeks post-procedure. Degree of facet joint degeneration was evaluated via MRI. Patients were categorized into two groups: low-grade facet joint degeneration group (group 1) and high-grade facet joint degeneration group (group 2).

**Results:**

A total of 147 patients were included in the study. NRS scores were significantly higher in group 2 compared to group 1 at the 3rd week follow-up. Treatment success, defined as a ≥ 50% reduction in NRS scores, was also significantly higher in group 1.

**Conclusion:**

Facet joint degeneration adversely impacts the treatment success of TFESI. A comprehensive evaluation of facet joint pathologies prior to procedure planning is imperative for optimizing treatment outcomes.

## Introduction

Epidural steroid injection is a widely used modality for the treatment of radicular pain caused by lumbar disc herniation [1]. Herniated disc generates pain through inflammation induced by chemokines, compression, and consequent ischemia of the nerve root [2, 3]. Transforaminal, caudal, and interlaminar applications are available; however, transforaminal epidural steroid injection (TFESI) is the most common and the most efficient technique facilitating direct delivery of the injectate to the targeted ventral epidural area [4]. The anti-inflammatory properties of steroids are pivotal to their mechanism of action. Steroids diminish the synthesis of numerous anti-inflammatory mediators, including cytokines and chemokines. Additionally, they enhance blood flow around ischemic spinal roots, promoting the removal of cytokines through the washout effect of the injectate [5–7].

The short-term efficacy of TFESI in treating radicular pain caused by LDH is well-documented. However, achieving favorable treatment outcomes in all patients remains a challenge, possibly due to various factors such as the duration and severity of symptoms prior to the procedure, nerve root compression, or the presence of facet tropism [8–10].

Facet joints are capsular synovial joints that connect superior and inferior articular processes and are located between the lamina and pedicle. Degenerative facet disorders such as osteoarthritis and hypertrophied superior articular process are the most common facet joint pathologies [11]. Each spinal segment comprises three weight-bearing structures: the intervertebral disc/vertebral body anteriorly and the two facet joints posteriorly. When there is a loss of structural integrity in one of these units, excessive weight-bearing is placed on the other [12]. The majority of the axial load is carried by the intervertebral discs; therefore, disc degeneration acts as a facilitating factor for the development of facet joint degeneration. Fujiwara et al. reported that facet osteoarthritis is seldomly observed in the absence of degenerative disc disease. Additionally, the most severely degenerated facet joints are typically found at levels with advanced intervertebral disc degeneration [13]. Hence, it is highly likely that a study population with lumbar disc herniation would also exhibit facet joint degeneration to some degree.

Moreover, pain stemming from degenerative facet disorders may manifest in referred areas such as the gluteal region, groins, thigh, and occasionally the distal legs. Additionally, degenerative facet pathologies can potentially induce radicular pain by compressing the neural foramen or dorsal root ganglion [14, 15]. As such, the presence of degenerative facet disease may impact treatment outcomes following TFESI by complicating the diagnostic process and potentially leading to residual pain resulting from untreated underlying conditions. To the best of the authors’ knowledge, no studies in the literature have analyzed the relationship between facet degeneration and TFESI treatment outcomes. Therefore, the present study is aimed at investigating the effect of facet joint degeneration on the treatment success of fluoroscopy-guided TFESI for single-level, single-root LDH. We hypothesize that treatment efficacy will significantly decline in patients with severe facet degeneration at or adjacent to the injection level.

## Materials and methods

Following approval of the local ethics committee, patients with radicular leg pain generated by single root and single level lumbar disc herniation who underwent transforaminal epidural steroid injection (TFESI) between January 2021 and September 2023 were retrospectively analyzed. In a university hospital setting, patients who were older than 18 years of age, who presented with unilateral radicular pain of at least 3 months’ duration, who had provided a pain score of 4 or higher on numeric rating scale (NRS), who had a single-root and single-level paracentral lumbar disc herniation on magnetic resonance imaging (MRI), and who had associated spinal root pressure were included in the study. Patients with bilateral or multiple level disc herniations, spinal stenosis, spondylolisthesis, or scoliosis; those who had undergone spinal surgery such as fusion or laminectomy; individuals showing signs of systemic or local infection; or with bleeding diathesis were excluded from the study. Patients lacking demographic data, pre-procedure clinical information, or post-procedure follow-up data in the medical records system were also excluded. Written informed consent was obtained from all patients included in the study.

## Radiological assessment

MRI records, performed within 6 months prior to the injection date, were retrospectively reviewed using the INFINITT PACS system. The assessment of facet joint degeneration was conducted on T2-weighted axial images following the method outlined by Weishaupt et al. [16]. This grading system comprises four grades: grade 0 indicates a normal facet joint space (2–4 mm), grade 1 denotes a narrowed joint space (less than 2 mm) and/or small osteophytes and/or slightly hypertrophied articular processes, grade 2 signifies a narrowed joint space and/or more pronounced osteophytes and/or hypertrophied articular processes and/or articular bone erosions, and grade 3 indicates a narrowed joint space and/or substantial osteophytes and/or extensive hypertrophy of the articular processes and/or severe bone erosions and/or subchondral cysts (refer to Appendix). Each patient underwent facet joint degeneration assessment at the level of disc herniation as well as one level below and one level above; the highest grade among the three levels was utilized for analysis. Patients were categorized into two groups based on the degree of degeneration: mild facet degeneration (grades 0 and 1) and severe facet degeneration (grades 2 and 3).

The degree of spinal nerve root compression was assessed using the grading system introduced by Pfirrmann et al. MRI T2-weighted imaging was employed for this assessment [17]. Grade 1 signifies slight contact between the disc and the nerve root, grade 2 denotes displacement of the nerve root with preserved fat tissue, grade 3 indicates obliterated periradicular cerebrospinal fluid or fat tissue, and grade 4 refers to morphologically disfigured nerve root.

## Procedure

Patients were transferred to the intervention suite and positioned prone with a pillow placed under the abdomen to achieve reduced lumbar lordosis. The lower back skin was cleaned three times with povidone iodine, and sterile draping was applied. A true anteroposterior (AP) image was obtained to determine the relevant level, after which the targeted foramen was visualized by positioning the C-arm at an oblique angle of 0–30° and a cranio-caudal angle of 0–15°. Initially, 3 cc of 2% prilocaine was injected into the skin and subcutaneous tissue. Subsequently, a 22-gauge, 3.5-inch spinal needle was advanced using intermittent fluoroscopic imaging via a coaxial technique. The immediate subpedicular area was targeted to reach the epidural space. The needle position was confirmed using a lateral view. One milliliters of contrast solution (300 mg I/mL iohexol) was administered to confirm epidural flow on the AP view prior to injecting a mixture of 10 mg dexamethasone, 1 mL of 0.5% bupivacaine, and 1 mL of saline. All injections were administered by an interventional pain specialist with 15 years of experience. Following the procedure, patients were transported to the recovery room to be monitored for 1 h. Patients without any complaints were discharged and scheduled for future outpatient clinic visits.

## Outcome measures

Pain intensity was assessed using the numeric rating scale (NRS-11) before the injection, as well as 1 h and 3 weeks after the procedure. Based on similar studies, a 3-week interval was selected as the earliest time for intervention, ensuring the corticosteroids’ effects are at their peak while avoiding compromise to the pituitary-adrenal axis [18]. A reduction of 50% or more on the NRS-11 compared to pre-procedure scores was considered a successful treatment outcome. Minimal clinically important change (MCIC) is defined as the smallest improvement in a patient-reported score that the patient perceives as beneficial, helping to exclude changes that are statistically significant but lack clinical relevance. In this study, a 2-point improvement on the NRS-11 scale was considered as MCIC [19].

## Statistical analysis

Statistical analysis was performed using SPSS version 27.0 software (IBM Corp.,

Armonk, NY, USA). Shapiro-Wilks test and Box Plot graphs were utilized in assessing the normal distribution of the quantitative data. Student t-test and one-way ANOVA test were used to evaluate normally distributed data while Mann–Whitney U test and Kruskal–Wallis test were used to compare data that were not normally distributed. For the comparison of qualitative data, Chi-square test, Fisher's exact test, and Fisher’s Freeman Halton test were employed. The results were evaluated at a 95% confidence interval, with significance set at p < 0.05.

## Results

**Fig. 1:**
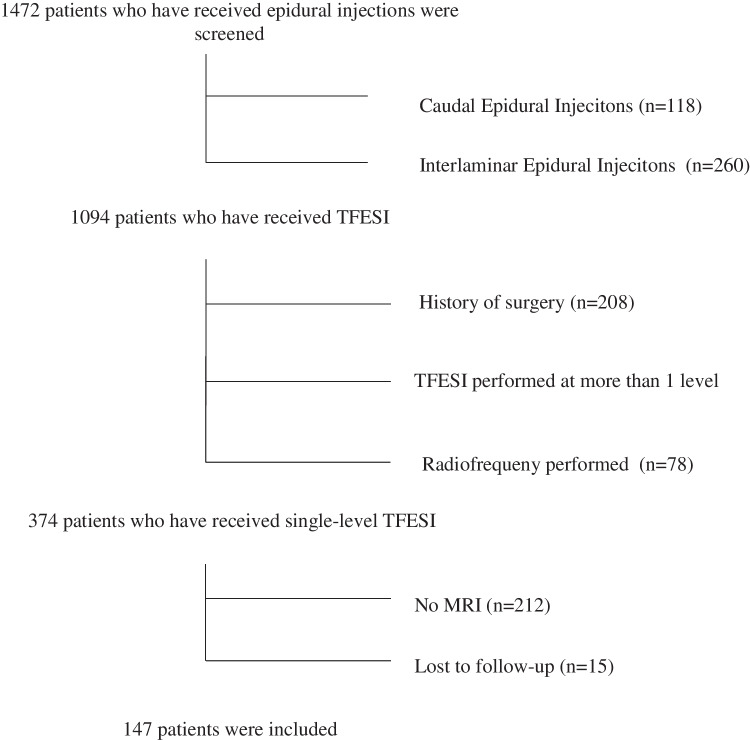
Flowchart for the selection process of patients included in the study

**Fig. 2:**
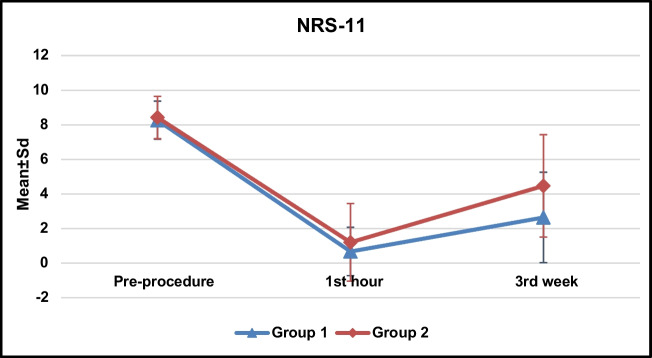
Temporal comparison of NRS-11 scores between groups

A total of 1472 patients who received epidural injections between January 2021 and September 2023 were screened. Patients who underwent caudal, interlaminar, or multiple-level epidural injections, as well as those with a history of spinal surgery or who had received radiofrequency treatment, were excluded. Of the 374 patients who received single-level TFESI 212 were excluded due to unavailability of MRI, and 15 patients were lost to follow-up. Consequently, data from 147 patients who met the inclusion criteria were analyzed (Fig. 1). Among them, 88 patients exhibited low-grade facet joint degeneration (referred to as group 1), while 59 patients demonstrated high-grade facet joint degeneration (referred to as group 2). Remarkably, there were no significant differences between the groups in terms of age, gender, body mass index, or duration of symptoms. Additionally, both groups had similar characteristics of LDH, including localization and severity of nerve root compression (Table 1). Both groups experienced a significant reduction in NRS-11 scores at the first hour and third week compared to pre-procedural scores. While there was no notable difference in NRS-11 scores between the two groups at the first hour, the NRS-11 scores at the third-week follow-up were significantly lower in group 1 (Table 2) (Fig. 2). Treatment success, defined as a ≥ 50% reduction in NRS-11 score following the injection, was observed in 94.6% of patients at the first hour and 68.7% at 3 weeks. There was no significant difference in treatment success between the two groups at the first hour. However, by the third week, treatment success was 78.4% in group 1, compared to 32% in group 2, which was significantly higher (*p* = 0.002) (Table 3). Treatment success with respect to minimal clinically important change (MCIC) was also significantly higher in group 1 at 3rd week follow-up; 77 (87.5%) patients in group 1 and 40 (67.8%) patients in group 2 demonstrated 2.5 or more units of improvement on NRS-11 scale (*p* = 0.004) (Table 4). To investigate the potential effects of nerve root compression on treatment success, changes in NRS-11 scores from pre-treatment to the 3rd week were compared across Pfirrmann grades within groups 1 and 2, but no significant differences were observed in either group (Table 5).

**Table 1:** Comparison of demographic and clinical features

		Group 1 (*n* = 88)	Group 2 (*n* = 59)	*p*
Gender	Female	49 (55.7)	37 (62.7)	^*a*^*0*.*396*
	Male	39 (44.3)	22 (37.3)	
Age (years)		49.14 ± 13.39 (48)	51.10 ± 13.02 (51)	^*b*^*0*.*379*
BMI (kg/m^2^)		27.13 ± 3.80	28.47 ± 5.07	^*b*^*0*.*088*
Side of lumbar disc herniation	Right	36 (40.9)	26 (44.1)	^*a*^*0*.*704*
	Left	52 (59.1)	33 (55.9)	
Duration of symptoms (months)		16.43 ± 26.61 (6)	27.12 ± 37.26 (12)	^*d*^*0*.*136*
Level of nerve root compression	S1	21 (23.9)	9 (15.3)	^*c*^*0*.*593*
	L2	1 (1.1)	1 (1.7)	
	L3	0 (0.0)	1 (1.7)	
	L4	12 (13.6)	8 (13.6)	
	L5	53 (60.2)	40 (67.8)	
	L6	1 (1.1)	0 (0.0)	
Severity of nerve root compression	Grade 1	9 (10.2)	5 (8.5)	^*a*^*0*.*742*
	Grade 2	35 (39.8)	19 (32.2)	
	Grade 3	31 (35.2)	25 (42.4)	
	Grade 4	13 (14.8)	10 (16.9)	

Values are presented as mean ± standard deviation (median) or *n* (%). ^a^Pearson Chi-square. ^b^Student-t test. ^c^Fisher Freeman Halton test. ^d^Mann-Whitney *U* test.

**Table 2:** Temporal comparison of NRS-11 scores between groups

		Group 1(*n* = 88)	Group 2 (*n* = 59)	*p*
Pre-procedure		8.26 ± 1.10 (8)	8.42 ± 1.22 (8)	^*d*^*0*.*411*
1st hour		0.67 ± 1.40 (0)	1.20 ± 2.24 (0)	^*d*^*0*.*205*
3rd week		2.64 ± 2.61 (2)	4.47 ± 2.96 (5)	^*d*^*0*.001**
	^*e*^*p*	*0*.001**	*0*.001**	

Values are presented as mean ± standard deviation (median). ^d^Mann-Whitney *U* test. ^e^Friedman test. **p < 0.01. *p < 0.05.

**Table 3:** Comparison of treatment success rates between groups

NRS-11		Group 1 (*n* = 88)	Group 2 (*n* = 59)	*p*
1st hour treatment success	No	3 (3.4)	5 (8.5)	^*f*^*0*.*268*
	Yes	85 (96.6)	54 (91.5)	
3rd week treatment success	No	19 (21.6)	27 (45.8)	^*a*^*0*.002**
	Yes	69 (78.4)	32 (54.2)	

Values are presented as *n* (%). ^a^Pearson Chi-square. ^f^Fisher exact test. **p < 0.01.

**Table 4:** Comparison of treatment success rates, with respect to the minimal clinically important change

NRS-11		Group 1 (*n* = 88)	Group 2 (*n* = 59)	*p*
3rd week minimally clinically important change	No	77 (87.5)	40 (67.8)	^*a*^*0*.004**
	Yes	11 (12.5)	19 (32.2)	

Values are presented as *n* (%). ^a^Pearson Chi-square. **p < 0.01.

**Table 5:** Comparison of NRS-11 scores with respect to nerve root compression (Pfirrmann grading)

		Grade 1	Grade 2	Grade 3	Grade 4	*p*
Group 1 (*n* = 88)	*n*	9	35	31	13	
	Pre-procedure	8.11 ± 0.93 (8)	8.03 ± 1.27 (8)	8.32 ± 0.98 (8)	8.85 ± 0.8 (9)	0.124
	Pre-procedure 3rd week change ∆	− 6.22 ± 1.86 (− 6)	− 5.46 ± 2.93 (− 6)	− 5.55 ± 3.03 (− 6)	− 5.85 ± 2.03 (− 6)	0.961
Group 2 (*n* = 59)	*n*	5	19	25	10	
	Pre-procedure	9.2 ± 0.84 (9)	8.32 ± 1.29 (8)	8.4 ± 1.22 (8)	8.3 ± 1.25 (8)	0.487
	Pre-procedure 3rd week change ∆	− 3.8 ± 1.92 (− 4)	− 3.63 ± 3 (− 4)	− 4.04 ± 3.16 (− 4)	− 4.4 ± 2.41 (− 4.5)	0.933

Kruskal Wallis test

Values are presented as mean ± standard deviation (median)

## Discussion

In this study, our objective was to examine the impact of facet joint degeneration on the treatment success of single-level unilateral TFESI. Confirming our hypothesis, treatment efficacy was significantly decreased in patients with severe facet degeneration. We observed that both the 1st-hour and 3rd-week NRS-11 scores were significantly lower compared to pre-treatment scores for all patients. However, during the third-week follow-ups, the improvement in NRS-11 scores was more pronounced in the group with low-grade facet joint degeneration than in the group with high-grade facet joint degeneration. Additionally, treatment success at the third week was significantly higher in the low-grade facet joint degeneration group (78.4%) compared to the high-grade facet joint degeneration group (54.2%). To the best of our knowledge, this study represents the first investigation into the effects of facet joint degeneration on TFESI treatment success.

Lumbar facet joint pain typically constitutes a distinct clinical entity that might be originating from any anatomical component that is vital to both function and architecture of facet joints such as joint capsule, hyaline cartilage, and synovium [15]. With a prevalence exceeding 50%, facet joint pain can manifest in areas such as the groin, flank, upper lateral thigh, lower lateral leg, and, in rare cases, the foot, potentially overlapping with dermatomal pain caused by lumbar disc herniation [20]. Adding complexity to diagnosis, facet joint pathologies can also give rise to radicular leg pain through spinal or foraminal stenosis. Conditions such as hypertrophied superior articular processes, osteophyte formation, or cysts can lead to narrowing of the foramen and induce radicular symptoms [21]. Yoo and Kim proposed that these structures may compress the dorsal root ganglion, triggering radicular pain. In such cases, particularly when nerve root compression induced by herniated disc is also present, pinpointing the origin of radicular pain may prove challenging [12]. Although our study included patients with documented paracentral lumbar disc herniation and nerve root compression, it is conceivable that foraminal stenosis or compression of the dorsal root ganglion resulting from concurrent facet joint degeneration contributed to the less favorable outcomes observed in the severe facet degeneration group.

As a rule of thumb, patients undergo a comprehensive history-taking process including the localization of pain (axial vs. radicular) and a thorough physical assessment including facet loading test prior to TFESI procedure. Complaints of axial pain, tenderness upon palpation of facet joints, or positive facet loading tests therefore lead the physician to consider facet pathology and performing a medial branch block which is the gold standard for facet joint pain. The patients of the current study exhibited radicular pain and yielded negative results for facet joint provocation tests. However, patients may still have had unreported axial pain to some extent probably masked by more prominent radicular pain. Therefore, residual axial lumbar pain following the procedure could be another explanation for the less favorable treatment outcomes in patients with severe facet joint degeneration. Moreover, despite careful selection of the study population based on nerve root compression, accompanying somatic pain originating from spinal structures such as intervertebral discs, facet joints, ligaments, and sacroiliac joints may have been present. It has been demonstrated that noxious stimulation of these structures can produce referred pain in addition to back pain [22, 23]. The mechanism of lumbar somatic referred pain does not involve nerve root stimulation; instead, it is related to the convergence of afferent nerve endings on second-order neurons that also receive neural input from the areas of the lower back and lower limb [24]. This mechanism can be considered another confounding factor in determining the origin of low back and lower extremity pain.

Therefore, it is plausible that patients experienced improvement in lower extremity pain while their axial pain persisted. Although pain severity in general was assessed using the NRS-11 scale, levels of axial and radicular pain were not queried separately. Significant improvement in radicular pain may have been underestimated in a single global NRS-11 assessment due to residual axial pain. Similarly, Manchikanti et al. investigated the effects of epidural steroid injections in a systematic review and concluded that the level of evidence was poor for axial pain but good for lumbar radicular pain [25]. Additionally, Parr et al. studied the effect of caudal epidural steroid injections and reported superior evidence for the disc herniation group compared to the axial pain or discogenic pain group [26]. Based on our results, we hypothesize that patients with severe facet degeneration may have responded to TFESI to some extent; however, accompanying axial pain induced by facet joint degeneration, which has been shown to be more resistant to epidural steroid injections, likely contributed to the less favorable outcomes observed in this group.

Pain relief following TFESI has been reported to range from 26 to 70% in various studies [27, 28]. This variability could be attributed to numerous factors such as techniques used, assessment parameters, outcome measures, and characteristics of the study population [29]. Identifying predictive factors that can influence TFESI treatment outcomes is crucial for tailoring the procedure for a more targeted patient group. For instance, Ekadahl et al. reported that a short duration of pain prior to injection, high-grade nerve compression, and younger age were associated with better treatment outcomes, while Lee et al. found young age to be predictive of less favorable responses [30, 31]. Factors such as facet tropism and vitamin D deficiency have also been linked to less favorable TFESI outcomes [7, 8]. McCormick et al. further reported that higher pre-procedure pain scores were associated with increased TFESI treatment success [32]. In our study, the two groups did not exhibit significant differences in terms of gender, body mass index, age, duration of pain, severity of nerve root compression, and pre-procedural pain scores. This allowed us to evaluate the sole effect of facet joint degeneration. We believe this is the first study to identify a link between facet joint degeneration and decreased TFESI success rates.

## Limitations

The primary limitation of this study is its retrospective design. For example, the absence of separate assessments for axial and radicular pain represents a significant constraint, as unreported axial pain may have been masked by more prominent radicular pain. This residual axial pain could explain the less favorable treatment outcomes observed in patients with severe facet degeneration and highlights the need for distinct evaluations of axial and radicular pain in future studies. Additionally, while facet joint degeneration was assessed and categorized into grades, its impact on spinal canal and foraminal stenosis was not directly evaluated. Stenosis severity may independently influence symptom intensity and treatment outcomes, particularly given the higher likelihood of concurrent stenosis in patients with high-grade facet joint degeneration. Furthermore, the short follow-up period limited the ability to evaluate long-term outcomes. Lastly, the absence of functional outcome measures prevents the results from being interpreted from a functional perspective.

## Conclusion

Facet joint degeneration correlates with less favorable outcomes of TFESI treatment. Given the complex nature of low back pain and the presence of multiple potential pain-generating structures, facet joint pain should be considered as an alternative underlying cause when TFESI fails to alleviate pain. Further large-scale, prospective studies with longer follow-up periods are needed to fully decipher the effects of facet joint degeneration on TFESI treatment success.

## Data Availability

The data that support the findings of this study are available from the corresponding author upon reasonable request.

## Appendix

See Table 6

**Table 6:** Criteria for grading osteoarthritis of the facet joints

Grade	Criteria
0	Normal facet joint space (2 ± 4 mm width)
1	Narrowing of the facet joint space (< 2 mm) and/or small osteophytes and/or mild hypertrophy of the articular process
2	Narrowing of the facet joint space and/or moderate osteophytes and/or moderate hypertrophy of the articular process and/or mild subarticular bone erosions
3	Narrowing of the facet joint space and/or large osteophytes and/or severe hypertrophy of the articular process and/or severe subarticular bone erosions and/or subchondral cysts

Weishaupt D, Zanetti M, Boos N, Hodler J. MR imaging and CT in osteoarthritis of the lumbar facet joints. Skeletal Radiol. 1999;28(4):215–219. 10.1007/s002560050503
